# Impact of customer participation in value co-creation on customer wellbeing: A moderating role of service climate

**DOI:** 10.3389/fpsyg.2022.877083

**Published:** 2023-01-17

**Authors:** Xiaofang Yi, Junaid Ul Haq, Shehzad Ahmed

**Affiliations:** ^1^Business School, Wuchang University of Technology, Wuhan, Hubei Province, China; ^2^Business School, University of International Business and Economics, Beijing, China; ^3^Faculty of Management Sciences, Riphah International University, Faisalabad, Punjab, Pakistan; ^4^Department of Business Administration, DCC King Fahd University of Petroleum and Minerals, Dhahran, Saudi Arabia

**Keywords:** value co-creation, resilience, customer wellbeing, service climate, customer participation

## Abstract

The purpose of the study is to investigate consumer wellbeing because of consumer participation, value co-creation, and customer resilience. This research identified the interaction effect of service climate in the presented context. The data were collected from 490 hotel customers. The structural equation modelling technique was used to observe the hypotheses testing. Participants of the study positively supported the impact of customer participation on customer wellbeing directly and indirectly. Moreover, results showed the partial mediation of customer resilience and value co-creation between customer participation and customer wellbeing. Service climates strengthen the relationship between customer participation and resilience and value co-creation. Theoretical and practical implications have also been added.

## 1. Introduction

Nowadays, service dominancy is the most concerned topic for researchers and practitioners (Yi and Gong, 2013). Successful service delivery and value co-creation depend on customers and other stakeholders such as employees, suppliers, and advisors (Nadeem et al., 2021). Similar researchers identified that customers participate in service delivery and value co-creation equally as the employees. In addition, value co-creation has emerged as a significant strength to gain a competitive edge in the competitive environment (Opata et al., 2021). Similar researchers argue that both employee and consumer behaviour collectively generate value. Value creation is the central aspect that leads the customer towards behavioural outcomes (Zhao, 2021). In addition, organisations focus on the betterment of the consumer and pay more attention to their wellbeing. Because of that customer wellbeing is the keenest aspect that leads the organisation towards success (Huang and Lin, 2021). Most organisations focus on developing customer wellbeing to retain their customers and keep them loyal (Currie et al., 2012).

Furthermore, customers’ resilience is the uttermost aspect that tweaks a customer towards wellbeing. Employee resilience is the employee’s capacity to recover from challenging and complex scenarios (Huang et al., 2019). The intricate and complicated procedures concerning the employee can be the service climate where a customer receives its services. Service climate is the most influential regulating aspect which influences service delivery. Previously, Yi and Gong (2013) dictated the importance of customer value co-creation because value creation is the most considered aspect for every service organisation to conceptualise their goals into reality. In addition, this research tends to answer the following queries: How does customer participation, directly and indirectly, regulate customer wellbeing? How does service climate interact with the relationship among customer participation, employee resilience, and customer value co-creation?

While organising the originality of the present research, most previous studies (Huang and Suo, 2021; Shah et al., 2021) documented customer participation as the sole aspect of developing value co-creation. Researchers (Yi and Gong, 2013; Nan, 2021) argue that the value of co-creation depends not only on the customers but also on employee behavioural characteristics. Containing both aspects in a single study is essential to elaborate on the value of co-creation development. Most previous studies (Huang and Suo, 2021; Shah et al., 2021) documented customer participation as the sole aspect of developing value co-creation. Researchers (Zhang Y. et al., 2022; Yi and Gong, 2013; Nan, 2021) argue that value co-creation depends not only on the customers but also on one employee’s loyal behavioural characteristics. Containing both aspects in a single study is essential to elaborate on *t*-value co-creation development. Furthermore, most of the researchers documented employee resilience in different research settings such as Business-to-Business and COVID-19 (Luu, 2021; Carvalho and Alves, 2022), in the context of burnout (Fan et al., 2020; Tang and Blocker, 2022) in antecedents of paradoxical leadership (Franken et al., 2020). Still, no research highlights its influence on customer participation service delivery paradigm. In the given literature, most of the researchers documented the service climate concerning customer emotion (Kang and Hong, 2021) and empowerment of service quality in the hospitality industry (Pham Thi Phuong and Ahn, 2021), and identified the service climate as an independent aspect in the employee jobs satisfaction (Son et al., 2021). Hence, no research identified its moderating influence concerning customer participation, value co-creation, and employee resilience.

First, this research aims to conclude the influence of customer participation and employee citizenship behaviour on customer value co-creation; second, to illustrate the power of customer participation on employee resilience; and third, to know the interaction effect of service climate on the relationship among customer participation, customer value co-creation, and employee resilience.

This research contributes both theoretically and practically. This research concluded that customer participation is integral to value co-creation and possesses employee resilience. So, practitioners must increase customer participation; it will help create value and lead the organisation towards customer wellbeing. This research identified resilience as a vital aspect of the service environment influencing customer wellbeing. Moreover, service climate is a crucial part of developing customer value co-creation.

## 2. Literature review

**Figure 1:**
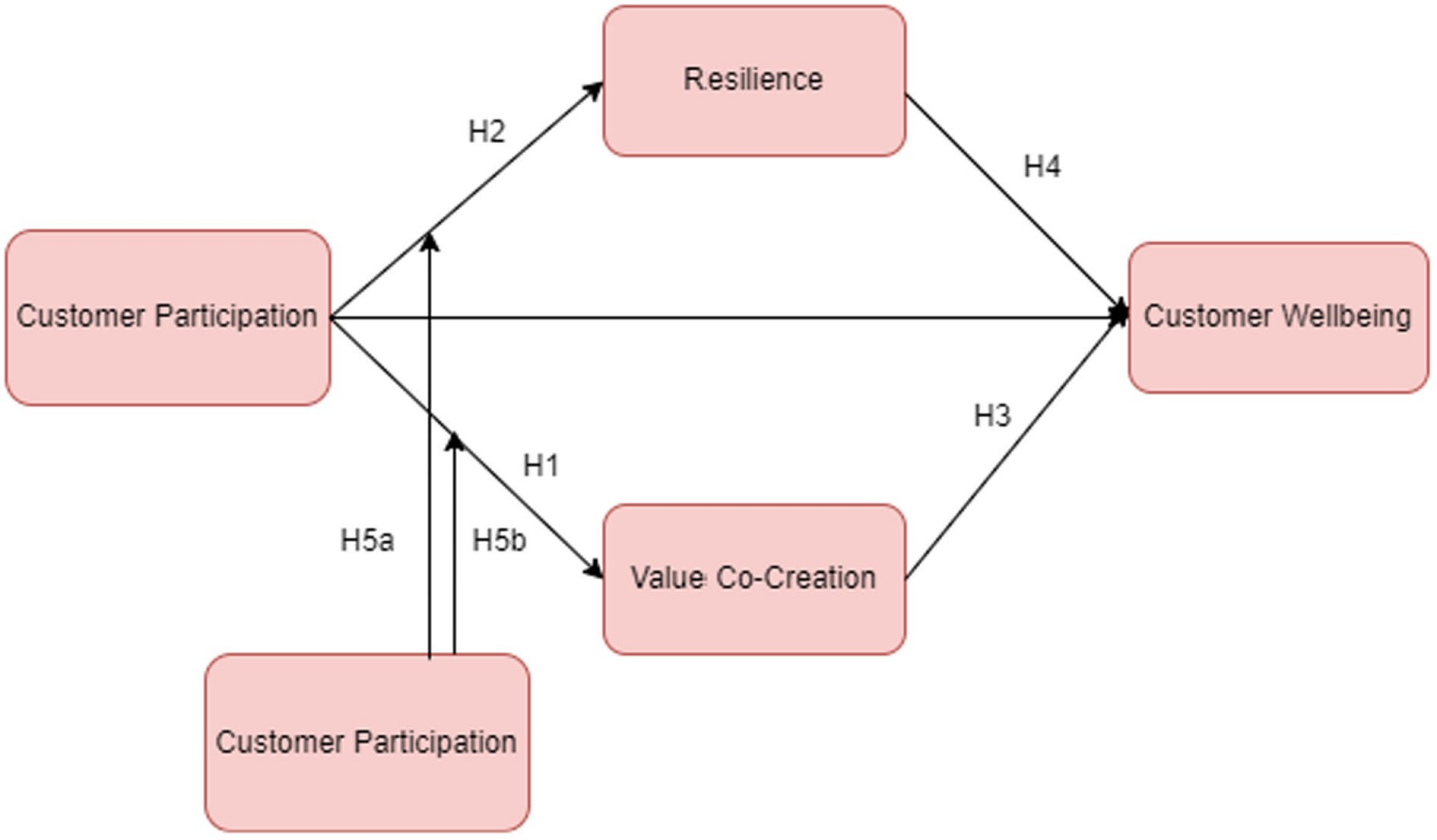
Conceptual framework.

This section will clarify the theoretical background and conceptual framework with strong backing from the literature. Initially, it will answer the research question (Figure 1).

### 2.1. Theoretical background

Social identity defines and evaluates one’s self-concept and how others will be treated and remembered (Tajfel et al., 1979). Social identity theory argues that inter-relations exist among the people of a community. This theory identifies that each group of individuals possesses the other individuals in a society (Brewer and Campbell, 1976). Also, Hoang (2022) and Ahmad et al. (2022) identify that service climate does not hold a single phenomenon; it comprises several factors such as interpersonal relationships and other environmental factors which develop a service climate. Aligning the concept of social identity theory with the service climate can be governed as the positive interpersonal relations rejoice desirable goals for the organisation (Hogg, 2016). The present research implication of this theory can be discussed as consumer and employee behaviours are influential elements of each other. Customer satisfaction is based on employee behaviour, and service delivery is based on customer responses. This research concluded that customer participation is an essential aspect of an employee’s customer value co-creation and resilience.

Wellbeing has come to fancy from Aristotle’s idea of eudemonia, which means prosperity or welfare. It is based on the comprehension of intellectual and moral intrinsic worth and the attainment of intellectual and physical competencies of oneself (Kristjánsson, 2016 as cited in Clement, 2010). Borgonovi and Pál (2016) define customer wellbeing as “a dynamic state characterised by customers experiencing the ability and opportunity to fulfil their personal and social goals. It encompasses multiple dimensions of consumers’ lives: cognitive, psychological, physical, social, and material. It can be measured through subjective and objective indicators of competencies, perceptions, expectations and living conditions.” The researcher identified that customer wellbeing encompasses both consumer and employee aspects (Casas, 2010); the present research concluded the employee aspect as customer value co-creation and employee resilience. Pollard and Lee (2003) addressed five key dimensions of wellbeing: physical, psychological, cognitive, social, and economic wellbeing. This research concluded all the dimensions as a whole construct.

### 2.2. Hypothesis construction

#### 2.2.1. Customer participation and customer co-value creation

Being customer-oriented only is not sufficient to compete in the market. Organisations must create value for the customer to retain and generate loyalty. Customer participation and customer value Co-creation are the two significant aspects. Both of these are interlinked to each other. Several authors support the validity of these two constructs (Engelman et al., 2017; Palma et al., 2019). Also, the researchers displayed that customer participation leads to organisational service delivery towards customer value creation for assistance; if a customer proactively participates during service delivery, then, fortunately, the quality of service becomes double. It will be valued to the money which is paid in its reward. Hence it is said as follows:

*H1:* Customer participation has a significant positive influence on customer value co-creation.

#### 2.2.2. Customer participation and employee resilience

Sometimes, customer participates in service delivery, but customer competency needs to facilitate the employee and organisation to create successful service delivery. The incompetency of customers creates a stressful environment, which influences an employee’s capabilities (Al-Hawari et al., 2020). Employee resilience is defined as the employee’s behavioural capabilities to overcome stressful and adverse job scenarios. Customer participation is always an essential and worthwhile aspect for every organisation; as much as the customer participates in the service delivery, it increases the possibility of success and satisfaction for both customer and the employee. Employees always try to overcome fear and stress to generate a positive vibe of service delivery. It can be affected by the incivility (Judge, 2009; Al-Hawari et al., 2020) of customer who participates in the service, so customer participation is a crucial aspect that influences employee resilience.

*H2:* Customer participation positively influences employee resilience.

#### 2.2.3. Customer value co-creation and wellbeing

Marketing literature suggests that wellbeing is an outcome of customer value Co-creation in the service dilemma (Guo et al., 2013). The research identified that customer engagement increases customer value co-creation by demanding more healthy activities to satisfy their demands. Satisfaction with these demands ultimately leads them towards wellbeing (Sharma et al., 2017). When a customer gets recommendations from the service employee, it will create more value for their knowledge and skills. The organisation engages customers to generate more engagement activities to increase participation. When a customer participates in the service delivery, it eases the employee to conclude a better understanding of the needs and wants of the customer (Sweeney et al., 2015; Zhang H. et al., 2022; Tahir et al., 2022). For instance, a medical examiner can treat the patient better when he provides information about the situation. It will create a better advisory for the customer and create value. This value creation leads the customer towards wellbeing; hence it is hypothesised as follows:

*H3:* Customer value co-creation has a positive influence on customer wellbeing.

#### 2.2.4. Employee resilience and customer wellbeing

Resilience is a strength-based concept that states one’s ability to bounce back, prosper, and thrive in stressful, fierce, and adverse situations (Benard, 1997; Cohen, 2013). Resilient employees show social competence, autonomy, and problem-solving skills and lead a purposeful and progressive interpersonal life (Benard, 1997, 2004). Resilience is an emerging development to respond to varied life situations (Masten, 2001). Experience with handling obstacles, coping strategies, and social support systems are the predictors of resilience (Rutter, 2006; Masten et al., 2008; O’Dougherty Wright et al., 2013). However, resilience can be deliberately learned and developed (Benard and Slade, 2009; Seligman et al., 2009; Cohen, 2013), so organisations can leverage the situation in their favour to increase employee performance and customer wellbeing. Studies have supported that resilience is protective against negative encounters in one’s life. All employees face risky encounters and adversity in their job portfolios, but resilience is a shield against vulnerability during these happenstances (Goldstein and Brooks, 2005; Friedli, 2009).

Consequently, employee resilience mitigates the adverse effects of these encounters linked with hoarded risk and distress, negatively related to customer wellbeing (Fergusson and Horwood, 2003). The organisation plays an essential role in developing adolescents’ employee resilience and preparing them for varied situations. Critical factors in promoting employee resilience include positive relationships with peers and customers, autonomy, self-independence in solving one’s problems, and self-efficacy (Masten et al., 2008). Hence it is said as follows:

*H4:* Employee resilience has a significant positive influence on customer wellbeing.

### 2.3. Customer participation and customer wellbeing

Customer service delivery refers to encouragement, modelling, reinforcement, and instructions to better their services (Hoover-Dempsey et al., 2005; Hoover-Dempsey and Sandler, 2005). According to Green et al. (2007), customer involvement is crucial and highly appreciated and supported in achieving employees’ desired emotional wellbeing.

According to Epstein (1995), sympathetic and supportive communiqué among peers, organisation, management, and employees who encounter that the customer may develop extended shared thoughtfulness about the service and its improved organisational support towards them, which is positively associated with customer mental health. Customer participation in service delivery plays a pivotal role in service success in organisations and increasing customers’ wellbeing. A substantial body of research supports this evidence that parents’ participation in service delivery positively affects various anticipated organisation-related attainments, for example, organisational performance and customer wellbeing (e.g., Fan and Chen, 2001; Hill and Tyson, 2009; Ma et al., 2016). Given the importance of customer participation in service delivery for its betterment, one should also keep in mind the moderation of this aspect. Some studies have found that intense behaviour of customer involvement can play an adversative role in developing customer wellbeing (Arnett, 2004). Considering this negative aspect of customer involvement in recent years, researchers are essential for identifying variables that can increase positive or beneficial customer participation in service delivery (Ma et al., 2016).

*H5:* Customer participation has a positive impact on customer wellbeing.

### 2.4. Moderating effect of service climate

Service is an organisation in which employees and customers work jointly and cooperatively to make it prodigious to facilitate customers’ service requirements and nurture social and emotional wellbeing (Durlak et al., 2011). This study focuses on service organisations’ psychosocial aspects, including norms, values, beliefs, attitudes, and expectations, that reinforce organisational life (Cohen et al., 2009; Aldridge et al., 2016, 2018).

Previous studies indicated that service climate is imperative in achieving organisational output and significantly develops customer wellbeing. Service climate revolves around building an effective relationship among all its stakeholders by creating an effective linkage among employee, consumer, and their peers, which incorporates a healthy and supportive culture to foster customer wellbeing. Another factor essential in developing customer welfare is creating a sagacity of connectedness. The evidence shows that when customers feel connected towards the organisation, customers ought to be achieving service outputs and self-efficacy. Also, these individuals are less likely to develop mental health issues and social disruption (McNeely et al., 2002; Bond et al., 2007; O'Brien and Bowles, 2013).

On the contrary, a weak connection with an organisation is linked with increased mental health issues and fruitless organisational life (Bond et al., 2007). The managerial climate approach involves the organisation working place of service delivery insightful. Service climate is connected with several outcomes, both during the job and over the life span, such as academics, social, psychological, and physical wellness (Felner et al., 2001; Cohen et al., 2009; Aldridge and McChesney, 2018). Organisations with a climate that is contrary to the ideal environment, somehow due to lack of management and managerial obliviousness about the importance of service climate on customer wellbeing (Cohen et al., 2009), designated this state as socially unjust and desecrated of customer rights of having a supportive service climate. In their recent review, Berkowitz et al. (2017) supported this notion. They stated that employee support, organisation safety, and connectedness are central to developing service climate support and student wellbeing construct. Hence, it is said as follows:

*H6a:* Service climate moderates the relationship between customer participation and customer wellbeing.

*H6b:* Service climate moderates the relationship between customer participation and employee resilience.

## 3. Methodology

This research intended to investigate the influence of customer participation on the development of customer co-creation and resilience and its impact on customer wellbeing. The researcher collected data from the hospitality industry, specifically from hotel industry customers, through a research questionnaire. The questionnaire comprised of three parts. The first part explains the overview of the research, the second part contains the demographics, and the third part includes the scales of variables. The data collection took 3 months before the current time.

### 3.1. Data collection and analysis

Questionnaires were distributed among the customers of the hospitality industry. The respondents of the present research belong to metropolitan cities. Researchers opted for both online and face-to-face questionnaires to collect the data. Google forms and face-to-face questionnaires were distributed to the respondents. The researcher adopted the research ideology (Hair et al., 2010) to conceptualise the data collection set. So, the researcher floated 500 questionnaires to minimise error. In this, 370 questionnaires were distributed through google forms, and the rest of the 130 questionnaires were distributed in person. Only 490 questionnaires are considered for the final data analytics. The rest of the questionnaires were excluded due to unfilled and non-serious respondents. After the data collection, descriptive statistics were applied in SPSS. To do this, the researcher attained the common method bias testing through Herman’s single-factor testing measures. In addition, AMOS software implied validity and reliability concerns in confirmatory factor analysis. At the same time, hypothesis testing was done in the structural equation model (SEM) in AMOS.

### 3.2. Measurements

The five-item scale of customer participation was adapted from Yim et al. (2012). Moreover, the five-item scale of customer value-co-creation was adapted from Chan et al. (2010). In addition, the nine-item scale of employee resilience was adapted from Näswall et al. (2019). A 16-item scale of customer wellbeing was adapted from Falter and Hadwich (2020). And the seven-item scale of service climate was adapted from Bowen and Schneider (2014). All the measurements were done by using the five-point Likert scale.

## 4. Results

The present research concluded the use of Gender, Age, and Education to measure the demographical impact of the respondents. The results of demographics are given in Table 1.

**Table 1:** Demographic.

Demographics	Frequencies	Percentage
**Gender**		
Male	321	71.3
Female	129	28.7
**Age**		
15–20	71	15.8
21–25	102	22.7
26 above	277	61.6
**Education**		
Highschool	196	43.6
College	191	42.4
Graduation	49	10.9
Masters	11	2.4
Post-graduation	3	0.7
**Marital status**		
Single	319	70.9
Married	133	29.1
**Hotel visit**		
Often	230	51.2
Sometime	190	42.2
Never	30	6.6

### 4.1. Reliability and validity

The reliability is measured by applying composite reliability (CR). All the CR values are within the acceptable range of 0.7–0.9, which illustrates a good result (Hair et al., 2014). Validity is measured by the implication of convergent and discriminant validity in confirmatory factor analysis by acquiring the methods of Fornell and Larcker (1981) and Hair et al. (2010). While convergent validity was assessed by considering the standard of average variance extracted (AVE), the values must be >0.5 for good measure. The results are given in Tables 2, 3. The model fit measures were concluded to check out the model’s fitness before performing the confirmatory factor analysis (CFA). The measures of model fit were comprised of CMIN/df 1.792, CFI 0.957, NFI 0.905, TLI, 0.953, IFI 0.957, RFI 0.900, and RMSEA 0.41; all the measures were within the thresholds.

**Table 2:** Convergent validity.

Variable	ITEM		FL	CR	AVE
Customer participation	PPC1	During my visit to the hotel, I actively share information I had with the employee who served me	0.823	0.894	0.630
	PPC2	I participate in a discussion about my case with the service personnel who served me at the hotel.	0.890		
	PPC3	While I am at the hotel, I told the service personnel what I know about my demands	0.876		
	PPC4	I make considerable effort to discuss my case with the service personnel at the hotel.	0.835		
	PPC5	I try my best to participate in my case at the hotel.	0.817		
Resilience	RES1	I effectively collaborate with others to handle challenges at work	0.883	0.946	0.715
	RES2	I successfully manage a high workload for long periods of time	0.900		
	RES3	I resolve crises competently at work	0.894		
	RES4	I learn from mistakes and improve the way I do my job	0.873		
	RES5	I re-evaluate my performance and continually improve the way I do my work	Deleted		
	RES6	I effectively respond to feedback, even criticism	0.880		
	RES7	I seek assistance at work when I need specific resources	0.877		
	RES8	I approach managers when I need their support	0.898		
	RES9	I use change at work as an opportunity for growth	0.856		
Customer wellbeing	STUW1	Are you happy with your ability to perform daily living activities?	0.794	0.947	0.530
	STUW2	Are you happy with your ability to work?	0.865		
	STUW3	Do you feel able to enjoy life?	0.846		
	STUW4	Do you feel optimistic about the future?	0.716		
	STUW5	Do you feel in control of your life?	0.784		
	STUW6	Do you feel happy with yourself as a person?	0.788		
	STUW7	Are you happy with your looks and appearance?	0.854		
	STUW8	Do you feel able to live your life the way you want?	0.714		
	STUW9	Do you feel able to grow and develop as a person?	0.816		
	STUW10		0.768		
	STUW11	Are you happy with your friendships and personal relationships?	0.815		
	STUW12	Are you comfortable about the way you relate and connect with others?	0.746		
	STUW13	Are you able to ask someone for help with a problem?	0.753		
Service climate	SC1	How would you rate the job knowledge and skills of employees in your business to deliver superior quality service?	0.859	0.912	0.603
	SC2	How would you rate efforts to measure and track the quality of service in your business?	0.863		
	SC3	How would you rate the recognition and rewards employees receive for the delivery of superior service?	0.881		
	SC4	How would you rate the overall quality of service provided by your business?	0.832		
	SC5	How would you rate the leadership shown by management in your business in supporting the service quality effort?	0.880		
	SC6	How would you rate the effectiveness of our communications efforts to both employees and customers?	0.826		
	SC7	How would you rate the tools, technology, and other resources provided to employees to support the delivery of superior quality service?	Deleted		
Customer value co-creation	CVC1	Customers let me know how to meet their needs better.	0.808	0.903	0.539
	CVC2	Customers tell me how to improve hotel services when he/she has new ideas	0.842		
	CVC3	Customers tell me about hotel service problems so that I can improve that	0.840		
	CVC4	Customers are willing to notify me the problem even if the problem does not affect themselves	0.819		
	CVC5	Customers will let me know if I give him/her good hotel service	0.844		
	CVC6	Even if the price error will benefit the customers, they will still remind me	0.862		

**Table 3:** Discriminant validity.

	CR	AVE	SCC	PPC	RES	STWB	CVC
SC	0.912	0.603	0.776				
CP	0.894	0.630	0.314	0.794			
RES	0.946	0.715	0.261	0.176	0.845		
CW	0.947	0.530	0.187	0.118	0.164	0.728	
CVC	0.903	0.539	−0.061	−0.014	0.001	−0.091	0.734

CP is customer participation, while CCV is customer value Co-creation. RES is resilience, and CW is customer wellbeing.

**Table 4:** Hypothesis testing.

SR	Hypothesis	SE	Accepted/Rejected
1	CP → CCV	0.128	Accepted
2	CP → RES	0.298	Accepted
3	CCV → CW	0.148	Accepted
4	RES → CW	0.228	Accepted
5	CP → CW	0.171	Accepted

CP is customer participation, while CCV is customer value co-creation. RES is resilience and CW is customer wellbeing.

### 4.2. Common method bias

The measure of common method bias depicts that the value of the percentage of variance must be <50%; and in the present case, it is 27%. The results show that the percentage of variance was 27.925, <50%, so it was within the acceptable range, the total extractions were 12.008, and the cumulative percentage was 27.925.

### 4.3. Hypothesis testing

The hypothesis testing was done in the Structural equation model SEM in Amos. The researcher applied the threshold of 0.05 by adopting the hypothesis technique (Bukhari et al., 2022). All the hypotheses got accepted (Table 4). At the same time, the researcher did the moderation and mediation measures in the respective later sections. The model fit measures were concluded to check out the model’s fitness before performing the Structural equation model. The measures of model fit were comprised of CMIN/df 1.522, CFI 0.944, NFI 0.885, TLI, 0.940, IFI 0.940, RFI 0.843, and RMSEA 0.33; all the measures were within the thresholds.

Results show that in the first hypothesis, the relationship between customer participation and value co-creation got significantly accepted by having a *p*-value of <0.05 and a value beta value of 0.128. In the second hypothesis, the relationship between customer participation and resilience was fully supported by having a *p*-value of <0.05 and a beta value of 0.298. In the third hypothesis, customer value co-creation significantly impacts customer wellbeing by having a *p*-value of <0.05 and securing a beta value of 0.148. Moreover, in hypothesis six, the relationship between resilience and customer wellbeing is also fully supported by attaining a *p*-value of <0; in this context, the beta value was 0.228. Therefore, customer participation shows a significant positive impact on customer wellbeing by achieving a *p*-value of <0.05; however, the beta value was 0.171.

### 4.4. Moderation analysis

**Figure 2:**
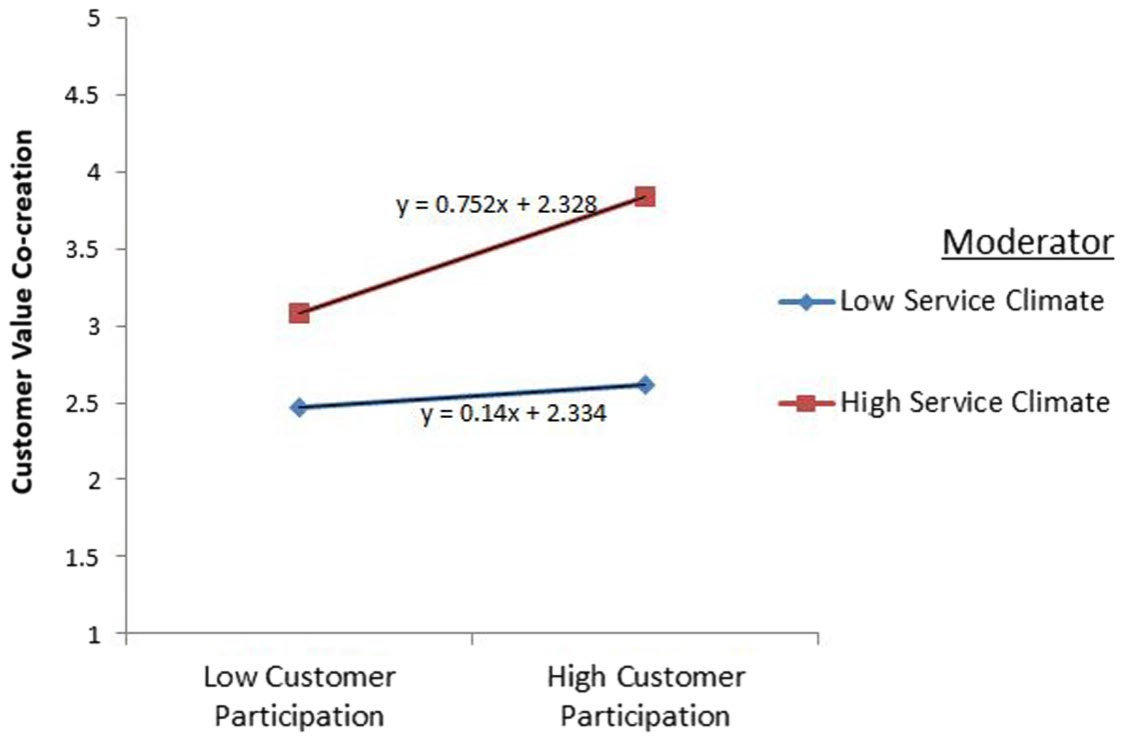
Service climate strengthens the relationship between customer participation and customer value co-creation.

**Figure 3:**
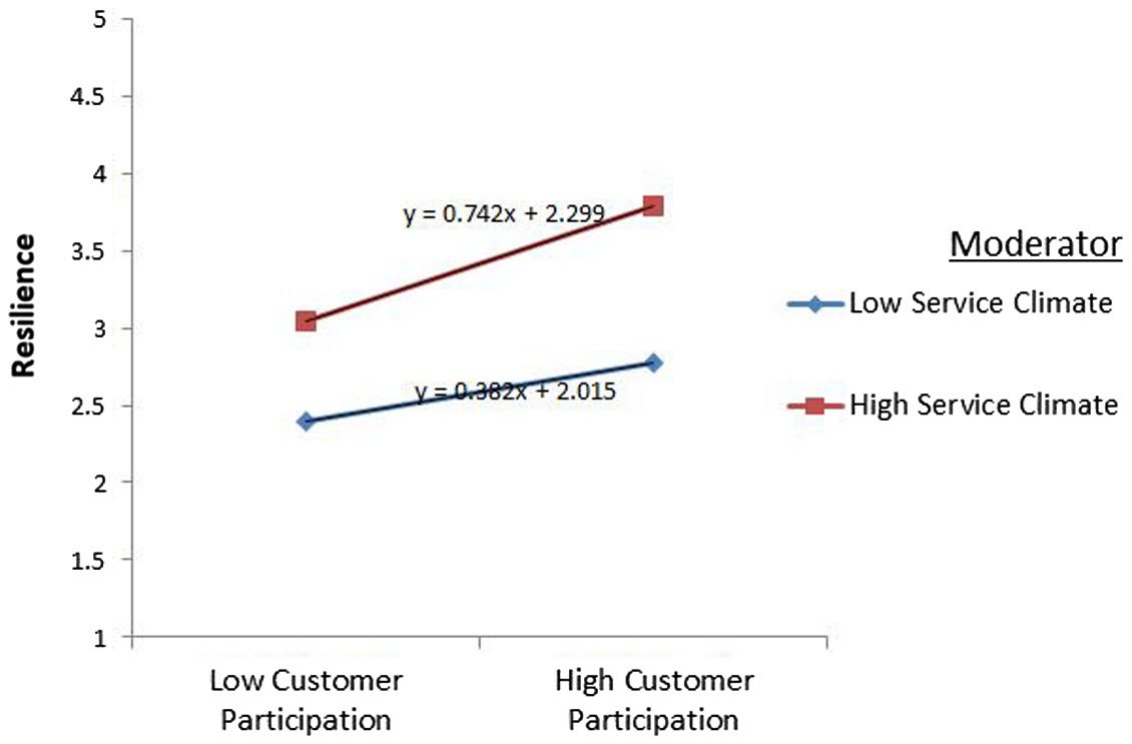
Service climate strengthens the relationship between customer participation and resilience.

Moderation measures were done in AMOS by implementing the partial SEM. The researcher adopted the technique of moderation measure, which was applied by Bukhari et al. (2022). The results of moderation measures are given in Figures 2, 3.

### 4.5. Mediation analysis

The researchers obtained the mediation measures in AMOS by applying the plugins in AMOS. The results show that both hypotheses got accepted by the threshold of 0.5. The results of the mediation analysis are given in Table 5.

**Table 5:** Mediation table.

Indirect path	Unstandardised estimate	Lower	Upper	Value of *p*	Standardised estimate
CP → CVC → CW	0.015	0.003	0.040	0.025	0.017*
CP → RES → CW	0.042	0.021	0.071	0.001	0.049***

CP is customer Participation, CVC is customer value co-creation, and RES is resilience.

In the first context, customer value co-creation shows full support by partially mediating the relationship between customer participation and wellbeing. The *p*-value was <0.05, which signifies the acceptability of this relation. However, the standardised estimates also show the influence of this relation by 0.017.

In the second context, employee resilience shows full support by partially mediating the relationship between customer participation and customer wellbeing. The hypothesis got fully accepted by achieving a *p*-value of <0.05. In addition to this, the value of standardised estimates was 0.049, which also shows the intensity of influence between the constructs.

## 5. Discussion

The results show significant results. In the first hypothesis, customer participation positively influences customer value co-creation, and the hypothesis got acceptance. Hence, this research signifies the conclusions of prior researchers (Yen et al., 2020). On the other hand, in the second hypothesis, customer participation positively possesses resilience, and the hypothesis got significant acceptance. Present research signifies the positive relationship between two constructs like past research (Simental et al., 2021). In addition, in the third hypothesis, the customer value co-creation positively impacts customer wellbeing; the present research complies with the truth of previous literature (Sharma et al., 2017). Furthermore, in the fourth hypothesis, resilience showed a significant positive influence on customer wellbeing. According to Partouche-Sebban et al. (2021), resilience is the most influential aspect of the development of customer wellbeing. In the fifth “A” hypothesis, value co-creation fully mediates the relationships between customer participation and customer wellbeing. Similarly, in the fifth “B” hypothesis, resilience positively mediates the relationship between customer participation and customer wellbeing. In addition, in the sixth hypothesis, discussing the moderation measures, Service climate strengthens both relationships.

### 5.1. Implications

This research implicates both theoretical and practical implications. The present study classified the implications as per construct.

#### 5.1.1. Customer participation

This research identified Customer participation as the essential construct of the marketing literature. This research clarifies that organisations must consider the impact of customer participation in their service portfolios. Managers must create an environment where customers can participate equally with the employee in service delivery. Customer participation leads the customer towards value co-creation, which means that customer participation increases the possibilities of value creation. In addition, customer participation helps increase employee resilience and cope with dynamic situations. In critical situations where the service delivery is quite unpredictable, it helps the employees to overcome such situations. This research conceptualises that managers must focus on customer participation because increasing customer participation will enhance customer wellbeing. Al-Hawari et al. (2020) identified customer participation as a crucial aspect of long-durable organisational interests.

Theoretically, this research replicates the authenticity of the marketing literature (Huang and Suo, 2021; Shah et al., 2021) by identifying customer participation as a possessor of the behavioural constructs. Specifically, in present scenarios, it possesses employee resilience and customer value co-creation; it is quite evident for research to create a complete conceptualisation of these constructs to develop the conceptualisation of customer behaviours antecedents.

#### 5.1.2. Customer value co-creation

This research created a significant conceptualisation of customer value co-creation, arguing that customer value creation is a crucial aspect of service delivery. This research concluded that value co-creation is based on customer participation. In today’s competitive environment, where every organisation pays more attention to getting a competitive advantage over other organisations, customer value co-creation creates customer wellbeing, which ultimately increases the competitive advantage for the service organisations. In addition, this research holds a potential implication for managers to consider the concept of value co-creation in long-term relations with customers and develop innovative ways to improve the organisation.

Theoretically, this research illustrates the concept of value co-creation on a broader ground, as Sharma et al. (2017) expressed the importance of value co-creation as a double-edged sword to incline customer participation and wellbeing. In addition, this research claims that researchers need to consider value co-creation as a mediating construct to develop consumer behavioural outcomes.

#### 5.1.3. Employee resilience

Present research identifies that service delivery is dynamic, and situations change every time. This nature of service sometimes depresses the employees, but employee resilience leads the employee to overcome such problems. This research argues that employee resilience influences customer wellbeing the most because as much as the employee has high resilience, it will increase customer wellbeing. Practitioners must focus on developing employee resilience through training and development programs to help them create more customer wellbeing. Although service situations are quite dynamic and vary from case to case, present research acknowledges that focusing on employee resilience can be beneficial to develop the desired outcomes and to achieve pre-set goals as prior research. Masten (2001) noted that employee resilience could help the employee to recover from such a situation where the customer shows some outrage and negative behavioural aspects. So, the present research also claims that it is beneficial to develop more positive consequences in scenarios where a customer is willing to participate in the service delivery.

Theoretically, this research accumulates the results of the previous study (Benard, 1997; Cohen, 2013) which suggested the importance of employee resilience as a core aspect of service delivery in the front-line employees’ encounters with consumers and their behavioural consequences. The present research concreted after the previous marketing literature. Current research illustrated that employee resilience could tweak not only consumer behaviour but also develops customer wellbeing. As suggested by O'Dougherty Wright et al. (2013), customer wellbeing can possess consumer behavioural aspects which ultimately cause positive consequences. Furthermore, this research contributes that resilience is the most integral construct to mediate positively between customer participation and customer wellbeing. It justifies the truthiness of previous research (Rutter, 2006; Masten et al., 2008), which also concluded the importance of employee resilience.

#### 5.1.4. Service climate

The current study illustrates that service climate is an incredible aspect influencing service delivery. Policymakers must develop service climates that ease customers and employees to create more value addition. Customer participation will get affected if the service climate is not appropriate. On the other hand, service climate positively relates to customer participation and employee resilience, which shows that even a highly resilient employee cannot play well in conditions where the climate is not suitable. Previous research (Cohen et al., 2009; Aldridge et al., 2016, 2018) signifies that service climate is the most influential aspect for achieving customer and organisational goals. Present research indicates that a positive service climate influences employee resilience positively. In this regard, managers must develop a better service climate to increase customer participation and employee resilience. Similarly, managers should focus on the service climate to establish more value co-creation.

Theoretically, previous research (Durlak et al., 2011) identified service climate as an integral part of the marketing literature. The present study put forward that service climate increases the quality of behavioural outcomes such as value co-creation and employee resilience. So, researchers must focus on the service climate as a crucial aspect of the marketing literature apart from the human resource literature. In addition, researchers must conclude that service climate tweaks the influence of the relationship among the predictors such as customer participation and customer value co-creation (McNeely et al., 2002; O'Brien and Bowles, 2013). Similarly, this also amasses the impact of customer participation and employee resilience, so researchers must convey this construct in the marketing literature and organisational fields. Most importantly, present research claims that social identity theory cannot only be applied to the consumer (Brewer and Campbell, 1976; Ahmad et al., 2022; Hoang, 2022), specifically the employee behavioural aspects; it also encompasses environmental aspects of the organisation. More specifically, service climate also includes the employees’ interpersonal relationships and professional relations with the consumer. So, researchers need to focus on the implementation of the social identity theory where consumer and employee interactions take place.

## 6. Conclusion and future research

Present research concluded that customer participation incorporates customer value co-creation and customer participation. In addition, it also includes customer participation and employee resilience as core aspects. In the case of customer participation and wellbeing, employee value co-creation positively mediates the relationship. Similarly, employee resilience also moderates the relationship between customer participation and customer wellbeing. In addition, customer participation creates customer wellbeing in dynamic service environments. Service climate is the central construct that moderates the relationship between customer participation and customer value Co-creation. It also influences the relationship between customer participation and employee resilience.

The present research is a cross-sectional study, so future researchers must longitudinally apply this research. In addition, the current research was conceptualised formally, so future researchers must informally replicate this research. Also, the present study was based on the service sector, so future research must be implemented in the product sector. With respect to this, the current research used survey methods to collect data. Still, future researchers must apply experimental designs by creating situations where customers and employees can participate in realistic conditions. In addition, experiments can be in terms of real products and service scenarios. In addition, future researchers can also arrange some interviews to keep the situations and data collection more realistic to increase customer participation in real means. However, this research can be implicated in the high-involvement service sectors such as banks, medical practitioners, educational platforms, and beauticians. Moreover, this research only concluded customer participation; future research must recognise the influence of employee behavioural characteristics such as employee citizenship behaviour and employee behavioural characteristics together with customer participation. This research was done in a developing country, so future researchers can implicate this research in developed countries by comparing the results.

## Data availability statement

The raw data supporting the conclusions of this article will be made available by the authors, without undue reservation.

## Ethics statement

This study involving human participants was reviewed and approved by the Ethics Committee of the Department of Management Sciences, Riphah International University Islamabad, Faisalabad Campus, Faisalabad, Pakistan. The participants provided their written informed consent to participate in this study.

## Author contributions

All authors listed have made a substantial, direct, and intellectual contribution to the work and approved it for publication.

## Conflict of interest

The authors declare that the research was conducted in the absence of any commercial or financial relationships that could be construed as a potential conflict of interest.

## Publisher’s note

All claims expressed in this article are solely those of the authors and do not necessarily represent those of their affiliated organizations, or those of the publisher, the editors and the reviewers. Any product that may be evaluated in this article, or claim that may be made by its manufacturer, is not guaranteed or endorsed by the publisher.
